# Numerical Analysis on the Dynamic Response of PVC Foam/Polyurea Composite Sandwich Panels under the Close Air Blast Loading

**DOI:** 10.3390/polym16060810

**Published:** 2024-03-14

**Authors:** Kaida Dai, Tao Jiang, Meng Zhao, Yuxin Xu, Xiaosong Zhao, Jiang Bian

**Affiliations:** 1 State Key Laboratory of Explosion Science and Safety Protection, Beijing Institute of Technology, Beijing 100081, China; 2 Beijing Institute of Space Long March Vehicle, Beijing 100081, China; bit_zhaom@163.com

**Keywords:** sandwich panel, PVC foam/polyurea composite, blast loading, dynamic response

## Abstract

This paper explores a novel structure aimed at enhancing its blast resistance performance by adding a layer of polyurea coating to the steel-PVC foam-steel sandwich panel. The response of 13 different arrangements of sandwich panels under explosive loading was studied using numerical simulation. The response process can be divided into three deformation stages: (1) Fluid-structure interaction; (2) Compression of the sandwich panel; (3) Dynamic structural response. The dynamic responses of the various sandwich panels to close-range air blast loading were analyzed based on the deformation characteristics, deflection, effective plastic strain, energy absorption, and pressure of the shock wave. The study draws the following conclusions: Reasonably adding a layer of polyurea to the traditional PVC foam sandwich panel can enhance its resistance to shock wave absorption, with a maximum increase of 29.8%; the optimal arrangement for explosion resistance is steel plate-PVC foam-polyurea-steel plate when the polyurea is coated on the back; and the best quality ratio between polyurea and PVC foam is 1:7 when the polyurea is coated on the front.

## 1. Introduction

In regions susceptible to explosives or potential acts of terrorism, extensive research has been conducted to enhance the impact resistance of engineering structures and mitigate explosion threats [1,2,3]. Because of its lightweight structure, easy production, and excellent mechanical properties, sandwich panels are widely used in civil and military fields, including the aerospace, navigation, construction, and automation industries. For this purpose, researchers have investigated the response of sandwich panels under high-intensity dynamic loading and explored various core materials, including honeycomb cores [4,5,6], corrugated-core [7,8,9], porous foam materials [10,11,12,13], metal lattices [14,15], balsa wood [16], and others. Porous foam materials’ low density and weight make them an ideal choice for lightweight applications, effectively reducing the overall weight of structures and decreasing energy consumption. Compared to honeycomb cores, porous foam materials have slightly inferior strength. However, they are easy to process and install and have lower production costs.

Previous studies [17,18,19,20,21] have primarily focused on investigating the mechanical behavior of several commonly utilized polymeric foam materials, such as polyurethane (PUR), polyethylene terephthalate (PET), polymethacrylimide (PMI), and polyvinylchloride (PVC), when employed as sandwich core layers. Among these materials, PVC foam composite sandwich panels exhibits characteristics such as highly specific strength and stiffness, high energy absorption efficiency, and strong buffering capability [22]. It can absorb blast energy by compressing and deforming the core layer under blast loading, thereby mitigating damage and destruction. A large number of studies have also shown that PVC foam, as the sandwich core layer, has excellent energy absorption characteristics [12,23,24,25,26,27]. For example, Hassan M.Z. et al. [26] studied the blast resistance performance of sandwich panels made up of PVC cores and aluminum alloy skins and demonstrated that the foam core absorbs more than half of the energy during an explosion. This finding is further supported by Ye’s study [26] on the high-speed dynamic response of PVC foam, which revealed that the stiffness of PVC material increases significantly as the strain rate rises. The modulus increases by 2.5 times compared to quasi-static conditions when the strain rate exceeds 1591 s^−1^ As a result, PVC foam exhibits excellent performance under blast loading. Besides, Zhou [24] showed that the blast resistance performance of PVC foam sandwich panels is influenced by factors such as core density, core thickness, panel thickness, and core shape. When the density of the PVC foam core increased from 80 g/cm^3^ to 250 g/cm^3^, the core provided more support to the front panel, resulting in a 36.1% reduction in deformation of the front panel and improved the blast resistance ability of PVC foam boards.

Higher core density increases the sensitivity of the foam core to tensile failure [27]. While keeping the core material unchanged, the strength of the sandwich structure can be enhanced by increasing the thickness of the front panel. When the surface density of the structure increased by 13.12%, the compression of the final core layer decreased by 24.35%, effectively reducing the deflection of the back panel [24]. Nevertheless, this will increase the weight of the structure, which is not conducive to lightweight design. As a result, adding sprayed or inserted polyurea layers in the sandwich panels effectively improves the mechanical strength of the structure [28,29,30,31], thereby enhancing its impact resistance. Polyurea exhibits characteristics, such as high internal friction and a significant strain rate effect during tension, transitioning from a rubbery state to a glassy state at high loading rates while dissipating a substantial amount of energy. Increasing the thickness of the front panel can effectively reduce the deflection of the rear panel as it has higher stiffness compared to the elastic intermediate layer. On the other hand, thickening the elastic interlayer minimizes additional mass while enhancing energy absorption and reducing structural deformation [32]. Inserting a thinner layer of polyurea in the sandwich panel can decrease the amplitude of compression waves transmitted to the core, thus reducing the deformation and strain of the board [22,33].

However, the optimal ratio and arrangement between the core and coating layers have not been addressed in previous studies. In this paper, simulation methods are employed to investigate the effects of various arrangements and mass ratios of PVC foam (with a density of 250 g/cm^3^) and polyurea under equal surface density conditions. A comprehensive analysis was conducted on the deformation, plastic deformation, and shockwave absorption of each sandwich panel. Subsequently, a quantitative evaluation criterion that combines three parameters was proposed to determine the optimal arrangement. This study can help clarify the strengthening effect of polyurea coatings on sandwich structures’ response and provide insights for future designs.

## 2. Materials and Methods

### 2.1. Validation of the Numerical Approach

**Figure 1:**
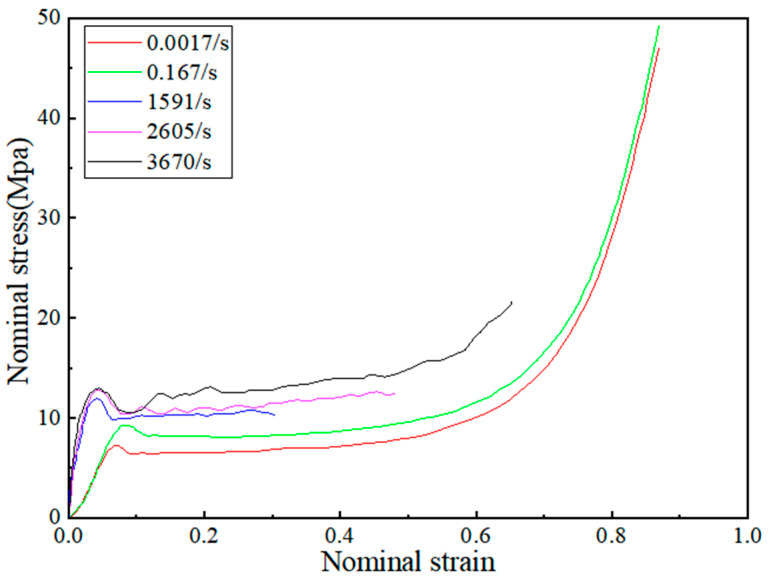
Stress-strain curves of PVC foam (H250) under uniaxial compression at different strain rates.

The PVC foam is orthotropic/transversely isotropic on a macroscopic scale, so the crushable foam material model was employed for the PVC foam core. The parameters of PVC foam were measured by Nan Ye [25] et al. through quasi-static and dynamic compression experiments, with a specimen size of Φ 30 mm × 10 mm. Table 1 [25] provides the parameters specific to PVC foam (H250) and the compressed data can be obtained from the curve depicted in Figure 1.

In order to remove deformed foam elements, an erosion criterion based on instantaneous geometric strain (with a threshold value of 2.0) was used [34]. The validity of the finite-element model and material parameters for PVC foam was confirmed by the comparison under 55 g TNT blast loading at 100 mm detonation distance in seven experimental conditions [24]. The loading conditions for all these scenarios are detailed in Table 1.

**Figure 2:**
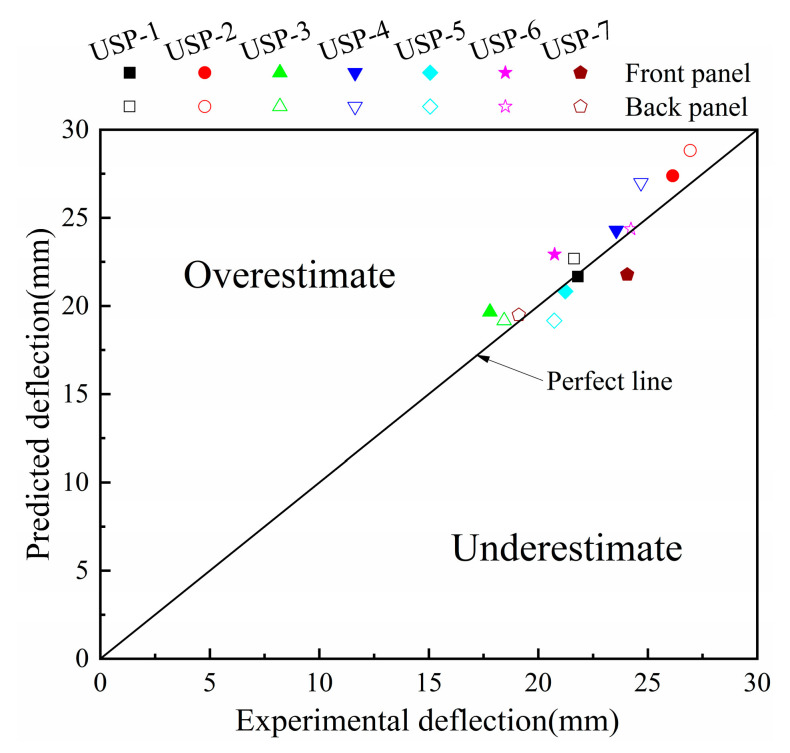
Comparisons between the experimental and numerical deflections under different conditions.

**Figure 3:**
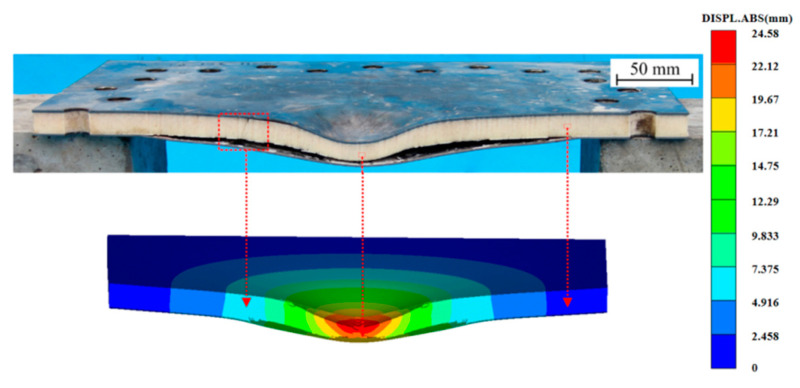
Comparison between the experimental and numerical deformation/failure modes of the panel USP-3.

The validity of the finite element model and material parameters of PVC foam has been confirmed through comparison with the experimental data from Zhou [24]. The simulation considers all conditions influenced by a 55g TNT explosion at a distance of 100 mm, as detailed in Table 2. The deflection predictions of the points in the panels were compared by using the experimental measurements, as shown in Figure 2 [24]. There was a good agreement between the numerical simulation results and the experimental results, with the points in the figures close to a perfectly matched line. In addition, a typical sandwich panel USP-3 was selected to verify the correlation between the numerical simulation and the experiment in terms of deformation/failure modes, as shown in Figure 3. It can be observed that the numerical simulation shows a significant local large deformation in the front panel and an overall deformation in the back panel. In addition, the compression and bending deformation of the core layer in the numerical simulation and the delamination failure between the core layer and the panel were also consistent with the experimental results. In summary, most of the details of the deformation/failure modes observed in the experiment are captured by the numerical simulation results.

The material model for polyurea, developed by the Naval Surface Warfare Center Carderock Division (NSWC-CD), adopted the Mooney-Rivlin hyperelastic material model. When the strain rate exceeded 300 s^−1^, the model’s coefficients were adjusted by using data which was published by Roland et al. [35] and Amirkhizi et al. [36]. The strain energy function of a hyperelastic material was expressed as a 2-parameter Mooney-Rivlin model, so it can be expressed as Equation (1):

(1) $$\psi =C_{10}\left({\bar{I}}_{1}-3\right)+C_{01}\left({\bar{I}}_{2}-3\right)+\frac{1}{d}{\left(J-1\right)}^{2}$$

**Figure 4:**
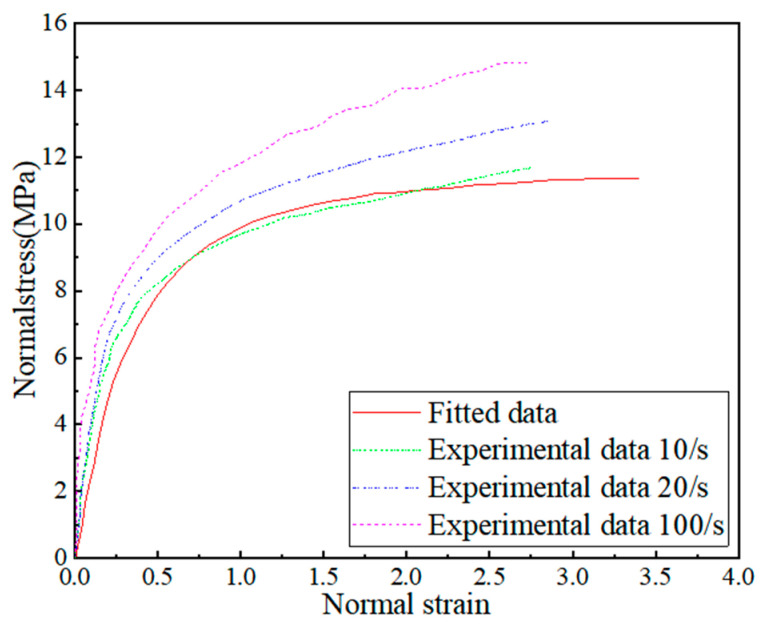
Results of high strain rate on polyurea and fitted data at 10 s^−1^.

The equation consists of several components for the material model of the polyurea. The constants $C_{10}$ and $C_{01}$ are determined by empirical observations, while ${\bar{I}}_{1}$ and ${\bar{I}}_{2}$ are the first invariant and the second invariant of the material. The determinant of the elastic deformation gradient is denoted by J, and parameter d represents non-compressed material properties. The values (C_10_ = 43.9 kPa, $C_{01}$ = 5568.1 kPa and d = 4 × 10^−7^ kPa^−1^) used in this study were obtained by fitting high strain rate tensile test data [36]. Figure 4 provides a visual representation of the test and fitting data [37]. In terms of material properties, polyurea had a density of 1020 kg/m^3^ and exhibited an adhesive strength of 80 MPa when it is applied to the steel panel. The failure model employed for polyurea involved the maximum main tensile strain criterion, with the failure strain of 1.63 [38]. This criterion is helpful to determine the point of failure of polyurea materials under tension.

**Figure 5:**
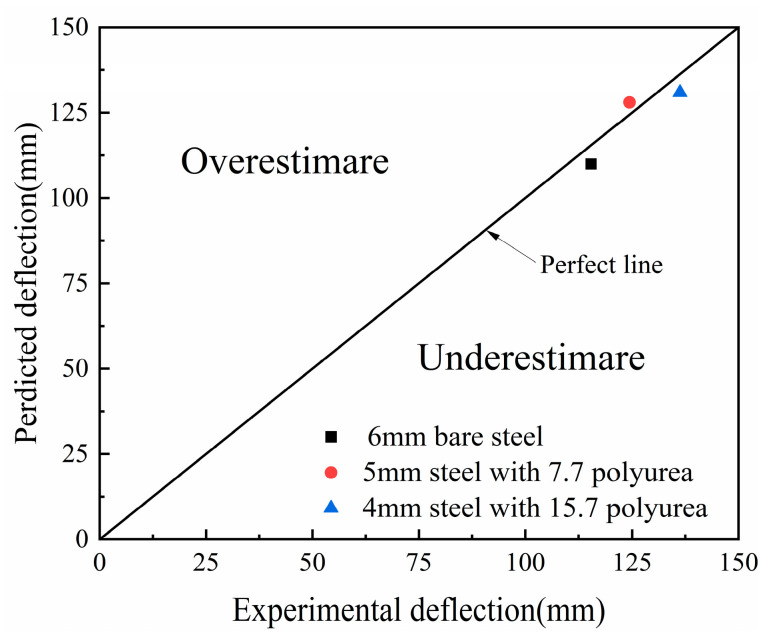
Comparison of simulation and experimental results of final deformation of polyurea-coated panels.

The numerical model of polyurea is compared and validated with the Auckland’s experiment [37], including three working conditions: (1) a 6 mm thick bare steel panel; (2) a 5 mm thick steel panel coated with thick 7.7 mm polyurea; (3) a 4 mm thick steel panel coated with 15.7 mm thick polyurea. The steel panels used in the model are Bluescope XLERPLATE 350 (Bluescope Steel. Bluescope steel 350 Xlerplate steel datasheet. Port Kembla: Bluescope Steel Limited, Port Kembla, Australia; 2009.®), and the explosive is Pentolite. The numerical simulation results were compared with the experimental results of Ackland, as shown in Figure 5. The numerical simulation accurately predicted the deformation of bare steel panels and polyurea-coated steel panel under air blast loading. Furthermore, the numerical model and material parameters reflected the mechanical properties of the steel panel and polyurea.

### 2.2. Modeling Geometry

**Figure 6:**
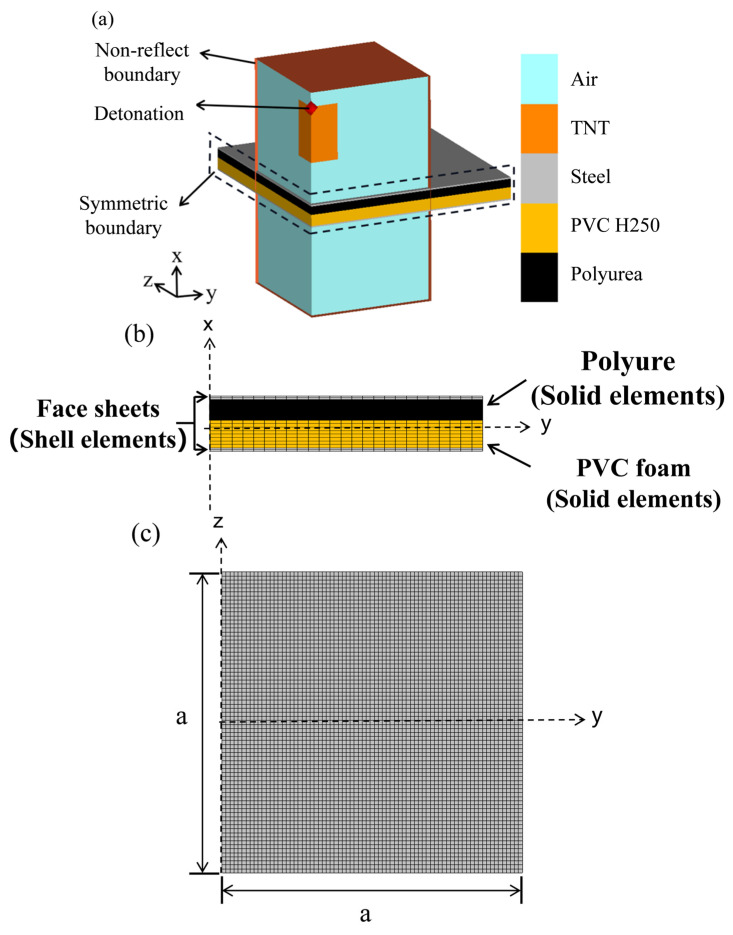
Structural diagram of DR-3: (**a**) Schematic of the sandwich panel; (**b**) Arrangement of the components in the sandwich panel; (**c**) In-plane discretization of the panels.

Considering the symmetry of the sandwich panel and the air blast loading, a quarter model was established to save computing resources, as shown in Figure 6. Symmetrical boundaries were set on the *Y* = 0 and Z = 0 planes, while the boundaries of the composite structure were completely fixed. When the blast distance was 50 mm, the panel was subjected to a localized loading that was primarily concentrated in the center. As a result, the air free field model covered an area of 200 mm × 200 mm in the center of the panel [7]. The outer surface of the air domain applied a free outflow boundary to simulate an infinite free field.

PVC foam core and polyurea were modeled using Lagrange solid elements, while air and explosives were modeled using multi-material Euler elements. And the front and back panels were modeled by using shell elements. In addition to the thickness direction, the Lagrange solid element was set to 1 mm, the remaining direction of the unit size was 2 mm, and the shell element was set to 5 mm.

The interaction between different parts of the sandwich panel was defined by using the penalty algorithm of frictionless contact. Sliding was utilized to determine the movement of contact nodes along the surface, and selecting the connection surface helped prevent node penetration. The shell thickness coefficient was set to 1. The bonding surface connection was used to connect adjacent parts. Except for the adhesion of polyurea to the substrate, the adhesion between other components was similar to that of epoxy resin adhesive, with tensile strength of 25 MPa and shear strength of 23 MPa [39]. The fluid–solid coupling algorithm was used between fluid Euler elements and structural shell elements or solid elements.

### 2.3. Material Properties

The PVC foam materials and polyurea materials have been presented in the second section of the previous text. The Johnson–Cook model was used to characterize the dynamic plastic behavior of 304 stainless steel. The dynamic flow stress ($\sigma_{y}$) was defined as a function of strain, strain rate, and temperature. The expression for the dynamic flow stress can be formulated as Equation (2):

(2) $$\sigma_{y}=\left[A+B{\left(\varepsilon_{p}^{eq}\right)}^{n}\right]\left[1+c\cdot \ln\left(\frac{{\dot{\varepsilon }}_{p}^{eq}}{{\dot{\varepsilon }}_{0}}\right)\right]\left[1-{\left(\frac{T-T_{r}}{T_{m}-T_{r}}\right)}^{m}\right]$$

The Johnson–Cook model considered material constants *A*, *B*, *n*, *c*, ${\dot{\varepsilon }}_{0}$ and *m*. The equivalent plastic strain $\varepsilon_{p}^{eq}$ and the equivalent plastic strain rate ${\dot{\varepsilon }}_{p}^{eq}$ are factors in the model. *Tr* represents the room temperature, while *T* represents the absolute temperature, and *T_m_* is the melting temperature of the material. In the case of 304 stainless steel, the Johnson-Cook parameters can be found in Table 3 [34,40]. To simulate panel fracture, a failure criterion based on equivalent plastic strain was employed. The failure strain is set to 0.4 [7].

Air material properties were described by using the ideal gas state equation. The relevant parameters were obtained from the ANSYS/Autodyn material library. To ensure standard atmospheric pressure, the initial internal energy of air was set to 206.8 kJ/kg [41]. For TNT explosives, its material properties were described by the JWL (Jones–Wilkins–Lee) equation of state. The values for the parameters associated with TNT were also obtained from the ANSYS/Autodyn material library. The ideal gas equation of state is suitable for describing the behavior of air, while the JWL equation of state is appropriate for modeling the properties of TNT explosives.

### 2.4. Computational Case Settings

To assess the various arrangement strategies of PVC foam and polyurea, as well as the impact of different mass ratios on the dynamic response of sandwich panels subjected to blast loading and shock wave attenuation, a series of sandwich panels with identical surface densities were designed. The panels had dimensions of 300 mm × 300 mm. In this study, an 80 g TNT explosive with dimensions of Φ 39.7 mm × 39.7 mm was employed to generate shock waves. The explosive charge was positioned at a fixed distance of 50 mm from the center of the front panel, which is defined as the blast distance.

Table 4 provides details on the panel names, the arrangement of the multi-layer core material and panels, and the corresponding thickness. The abbreviations used in Table 4 are defined as follows:

- DP—PVC Sandwich panels with different arrangement strategies of foam and polyurea
- DR—PVC Sandwich panels with different quality ratios of foam and polyurea
- F—PVC foam with a density of 250 kg/m^3^
- P—polyurea
- S—304 stainless steel

The DP panels included five distinct arrangement strategies, with a total thickness of 8 mm for PVC foam and 6 mm for polyurea. Within the DR panels, nine different sandwich panel configurations with varying mass ratios were included, maintaining polyurea foam in close proximity to the front panel and PVC foam near the back panel. DR-1 and DR-9 were utilized as reference panels for comparison, each consisting of a single sandwich material (either PVC foam or polyurea). For comparative analysis, it should be noted that DP-1 and DR-3 shared the same configuration, despite their different names.

## 3. Results

**Figure 7:**
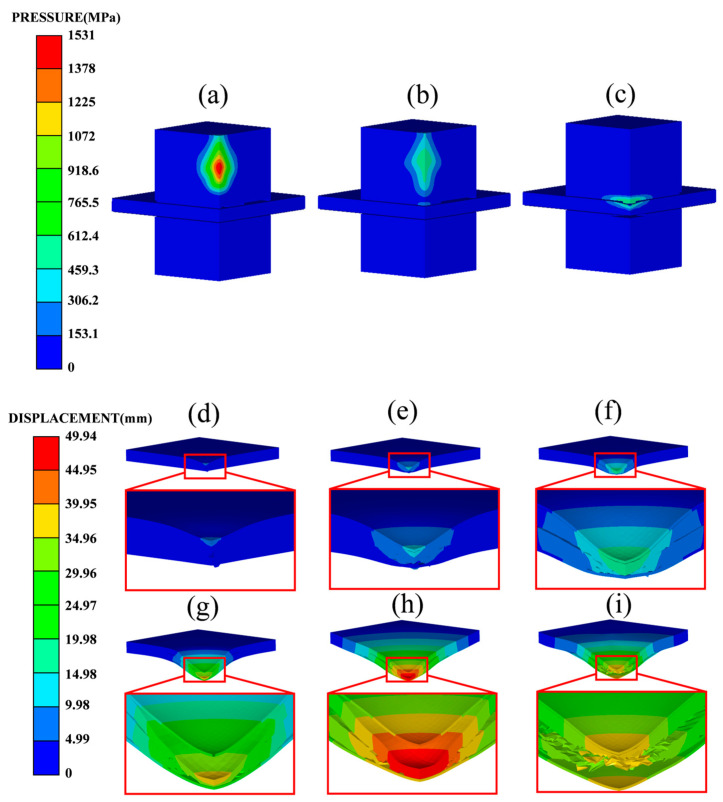
Typical processes of explosion product-structure interaction and panel deformation: (**a**) t = 9.3 μs; (**b**) t = 11.5 μs; (**c**) t = 20 μs; (**d**) t = 25 μs; (**e**) t = 35 μs; (**f**) t = 52.5 μs; (**g**) t = 120 μs; (**h**) t = 500 μs; (**i**) t = 1500 μs.

Figure 7 illustrates the response process of the DP-1 sandwich panel at a blast distance of 80 g TNT at 50 mm. In the beginning, the explosive products didn’t reach the sandwich structure (Figure 7a), then the volume of the explosive products continued to expand. And the shock wave continued to propagate outward; first, it hit the center of the front panel (Figure 7b). At this time, the stage of fluid-structure interaction began. The front panel obtained an initial velocity under the action of a shock wave (Figure 7c), while the core layer and back panel remained stationary. In the core layer compression stage, local indentation deformation was first formed in the central area of the front panel (Figure 7d), and then the front panel began to compress the core layer (Figure 7e), and the core layer was gradually densified. It can be observed that the degree of compression in the core decreased gradually from the central region to the outer region. Under the influence of its own bending resistance, tensile resistance, and the support force from the core, the front panel maintained decelerating motion. And the back panel underwent accelerating motion due to its own bending resistance, tensile resistance, and the pressure from the core. In the end of the compression phase of the core, the front and back panels reached the same velocity (Figure 7f). The panels then entered the phase of structural dynamic response under their own inertia (Figure 7g). In this phase, both the front and back panels started decelerating, and the front panel was decelerating more quickly. The front panel continued to compress the core, making it denser and expanding the compression range towards the outer region. The back panel decelerated to zero velocity under its own resistance and the cohesive force between the panel and the core, reaching its maximum deflection (Figure 7h). Subsequently, the sandwich panel entered an oscillation state (Figure 7i), until the kinetic energy gradually dissipated under plastic bending and stretching. The response of sandwich panels subjected to air blast loading can be described as three consecutive stages: the fluid-structure interaction stage, the core layer compression stage, and the structural dynamic response stage [27].

### 3.1. Analysis of Numerical Simulation Results of Sandwich Panels with Different Arrangement Strategies

#### 3.1.1. Analysis of Deformation Characteristics of the Panel

The degree of localization of panel deformation is closely related to the blast resistance capability of the sandwich panel. To investigate the influence of different arrangement strategies on the degree of localized panel deformation, the deflection surface data of the front and back panels under various working conditions were extracted and fitted by using Equation (3).

(3) $$w=w_{0}\theta^{\left(-\frac{x}{a}\right)}$$

**Figure 8:**
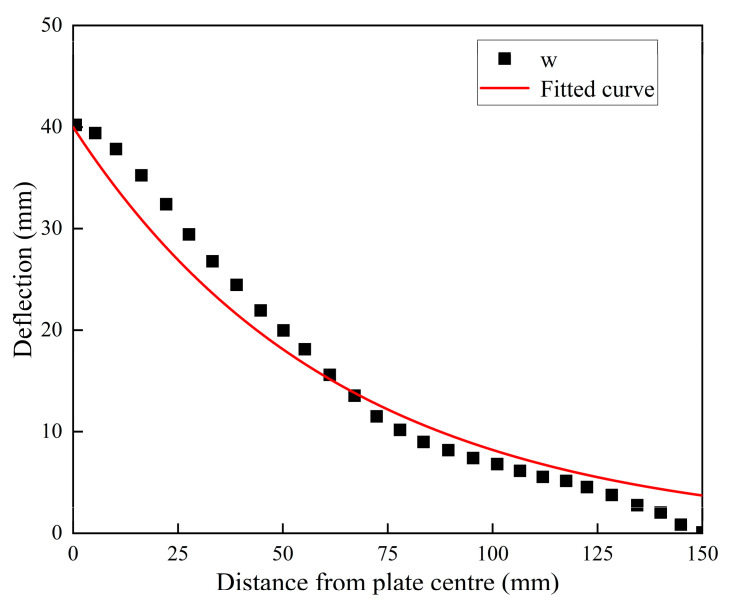
Deformation data and fitted curve of DP-1’s front panel.

In the formula, w represents the deflection of each point on the x-axis of the front panel, $w_{0}$ represents the deflection of the midpoint of the panel, 2a represents the side length of the board, and θ is the parameter that requires fitting. The fitting results for the front panel of DP-1 is shown in Figure 8, with a fitted R value of 0.968. The fitted θ values for the sandwich panels with different arrangement strategies are provided in Table 5.

The larger the θ value is, the stronger the localization degree of the panel is, and the weaker the localization degree of the panel is. The impact pressure of the panel with obvious localization characteristics was concentrated in the central area, which makes the panel more prone to yield and reduces the energy absorption potential of the core layer.

**Figure 9:**
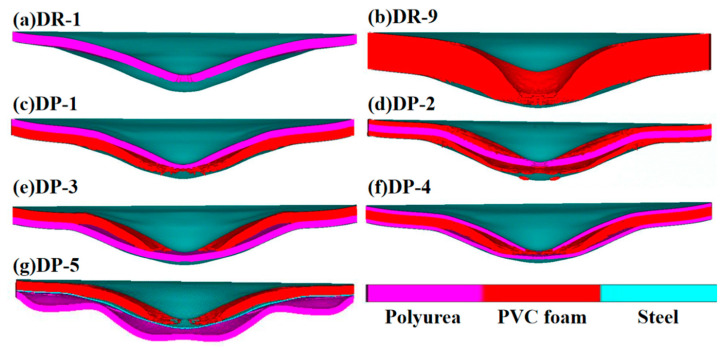
Cross-sectional view of sandwich panels’ deformation: (**a**) DR-1; (**b**) DR-9; (**c**) DP-1; (**d**) DP-2; (**e**) DP-5; (**f**) DP-4; (**g**) DP-3.

The θ values of DR-1, DP-1, and DR-4 of the reference panels coated with polyurea on the front panel were smaller than those of other arrangement strategies. Polyurea is a hyperelastomer that absorbs energy through overall tensile deformation, which changes the deformation characteristics of the front panel coated with polyurea and greatly weakens the localization degree of the front panel. The cross-sectional morphology of the final deformation of different panels is displayedAs shown in Figure 9. The reference boards DR-9, DP-2, DR-3, and DP-5 with PVC foam behind the front panel had large θ values, and PVC foam had excellent compressibility, which provided enough deformation space for the central area of the front panel and enhanced the localization degree of the front panel. Among the various sandwich panel arrangements, the reference board DR-9 exhibited a relatively weaker localization degree, as the excessively thick PVC foam offered strong bending resistance. On the other hand, the DP-2 arrangement, which had two layers of PVC foam, exhibited a higher degree of localization for the front panel. The polyurea coating on the PVC foam restricted its overall tensile deformation, resulting in a certain level of localization. For DP-5, the polyurea was sprayed on the outer side of the back panel. The compression waves reflected on the free surface and generated tensile waves, which caused widespread delamination and failure between the back panel and the polyurea. Consequently, the support provided by the polyurea to the back panel significantly decreased. The relatively thinner PVC foam in the center region of the front panel provided deformation space but offered little bending resistance. And it led to the strongest degree of localization for the front panel deformation in the DP-5 arrangement.

The θ values of the back panel were much smaller than those of the front panel. This indicated that large overall deformation remained the main characteristic of the back panel. Of the configurations, DP-2 and DP-5 had slightly larger θ values. When the polyurea was used as a sandwich between two layers of PVC foam or sprayed on the outer side of the back panel, it hindered the overall large deformation of both the back panel and the front panel. On the other hand, DP-3 exhibited the smallest value for the back panel. The thicker polyurea directly sprayed on the back panel weakened its localized characteristics.

#### 3.1.2. Panel Midpoint Deflection Analysis

**Figure 10:**
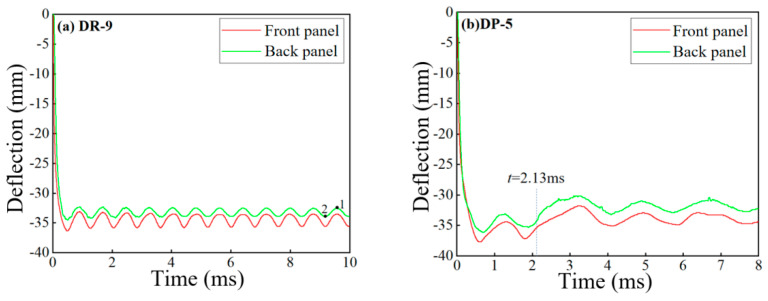
Deflection history curve at the midpoint of sandwich panels: (**a**) DR-9; (**b**) DP-5.

**Figure 11:**
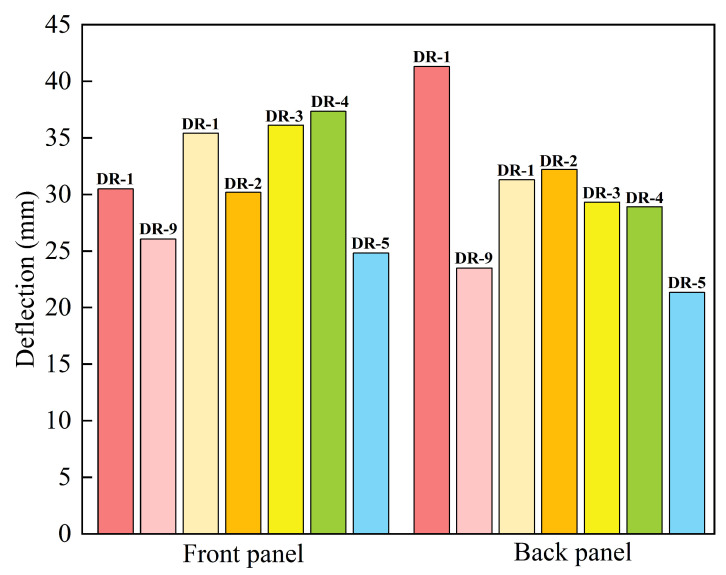
Midpoint deflections of sandwich panels with different arrangement strategies and reference panels.

The deflection at the midpoint of the panel is an important reference criterion for evaluating the blast resistance performance of the sandwich panel. Due to the long duration of panel vibration, the average value of the peak deflection at the midpoint of the panel (points 1 and 2 in Figure 10a) was taken as the final deflection. Unlike other sandwich panels, the deflection of the DP-5 panel at the midpoint suddenly decreased at 2.13 ms, as shown in Figure 10b, where the polyurea on the outer side of the back panel peeled off due to the tensile wave and rebounds. And it impacted the back panel. The deflections of the sandwich panels and the reference panels under different arrangement strategies are shown in Figure 11.

The deflection of the DR-1 front panel was 7.04% higher than that of the DR-9 front panel. The thicker polyurea support provided to the front panel restricted its movement, but also reduced the degree of localization. And this results in overall large deformation and greater deflection in the DR-1 panel. The deflection of the DP-1 front panel was 7.54% higher than that of the DR-1 panel. The appropriate thickness of the PVC foam core allowed for greater movement of the front panel, and increased the deflection of the DP-1 panel. Due to the support of the PVC foam, the DP-2, DP-5, and DR-9 front panels had a stronger degree of localization and smaller deflections.

**Figure 12:**
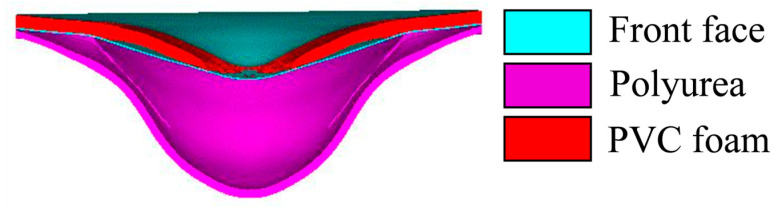
Cross-sectional view of DR-5 deformation.

Apart from DP-5, the localization degree of the back panels was similar, with overall large deformation being the main characteristic. The deflection of the back panel was mainly influenced by the cushioning effect of the core layer and the bending resistance. The DR-1 back panel had the highest deflection, 29.64% higher than the DR-9 back panel. Polyurea was not an effective cushioning material, and the DR-1 back panel still experienced a significant impact force. On the other hand, the PVC foam in the DR-9 panel absorbed and reduced the impact force by compression deformation, and provided better protection to the back panel. Additionally, the thicker PVC foam had strong bending resistance, which resulted in smaller deflection of the bonded back panel. DP-1, DP-2, DP-3, and DP-4 had the same core layer but different arrangement strategies, and their back panels had similar deflections, all of which were smaller than DR-1 but larger than DR-9. This indicates that the material and thickness of the core layer are the primary influencing factors on its cushioning capability, while the different arrangement strategies and layer numbers have a smaller impact on the deflection of the back panel. Among them, DP-4 had the smallest deflection, and the deflections of back panels of DP-1, DP-2, and DP-3 were 3.85%, 5.13%, and 0.69% higher than that of DP-4, respectively. The DP-5 back panel had the smallest deflection, 11.60% less than that of DP-4. This was caused by the larger deformation space provided by the polyurea spray on the outer side of the back panel. The compression wave reflected as a tensile wave at the free end, which caused the polyurea to detach from the back panel and undergo significant tensile deformation, as shown in Figure 12. The propagation of the tensile wave towards the back panel was blocked, and the elasticity of the polyurea further reduced the deflection of the back panel.

#### 3.1.3. Effective Plastic Strain Analysis of Panels

**Figure 13:**
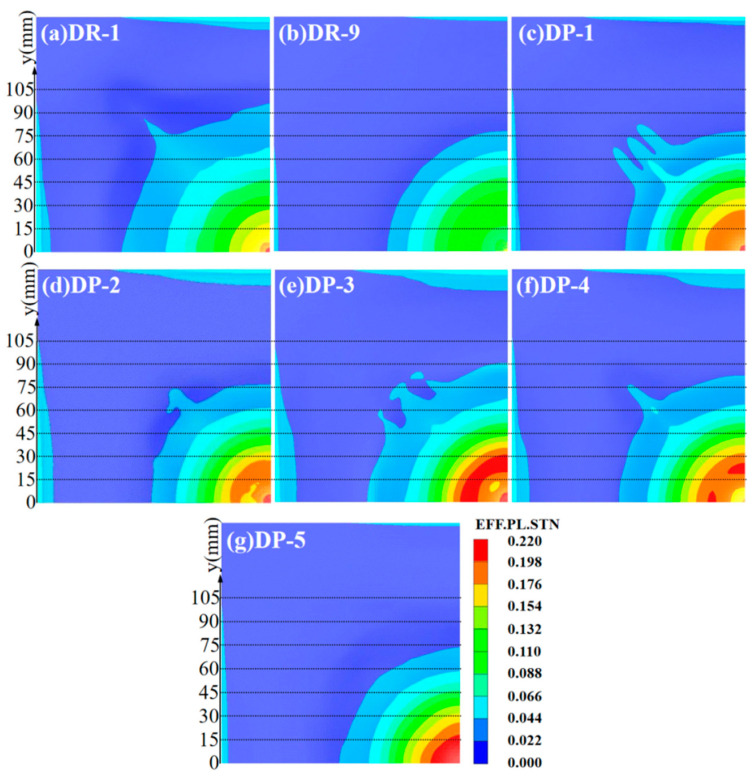
Effective plastic strain of front panels at t = 1000 μs. (**a**) DR-1; (**b**) DR-9; (**c**) DP-1; (**d**) DP-2; (**e**) DP-3; (**f**) DP-4; (**g**) DP-5.

The effective plastic strain of the panel can better reflect the blast resistance and structural strength of the sandwich panel, in comparison with deflection. The contour maps of effective plastic strain for the reference panels and the front panels of sandwich panels with different arrangement strategies are shown in Figure 13. Due to its higher degree of localization and smaller deflection, the DR-9 front panel had the smallest range of effective plastic strain which reached 0.044 (y = 78.07 mm), and the central region of the DR-9 front panel also had the smallest effective plastic strain. On the other hand, the weaker degree of localization in the DR-1 front panel results in the largest range of effective plastic strain, reaching 0.044 (y = 95.74 mm), and the central region had a smaller effective plastic strain than the front panels of sandwich panels with different arrangement strategies.

By comparing the y-values and areas where the effective plastic strain reached 0.176 and 0.198, we can quantitatively assess the blast resistance of the front panels with different arrangement strategies. The strain conditions in the central regions of the DP-1 and DP-2 front panels were similar, with y-values at 26.91 mm and 28.83 mm, respectively, where the effective plastic strain reached 0.176. And it indicates the best blast resistance. The DP-4 front panel had a y-value of 33.04 mm where the effective plastic strain reached 0.176, and a larger area where the effective plastic strain reached 0.198. It indicated a slightly weaker blast resistance. The DP-5 front panel had a y-value of 27.02 mm where the effective plastic strain reached 0.176, similar to DP-1, but a high y-value of 18.98 mm where the effective plastic strain reached 0.198. This indicated a weaker blast resistance than DP-4. The DP-3 front panel had the largest y-values of 36.923 mm and 30.904 mm where the effective plastic strain reached 0.176 and 0.198, respectively. This indicated the weakest blast resistance. The ranking of blast resistance for the front panels is as follows: DR-9 > DR-1 > DP-1 > DP-2 > DP-4 > DP-5 > DP-3.

**Figure 14:**
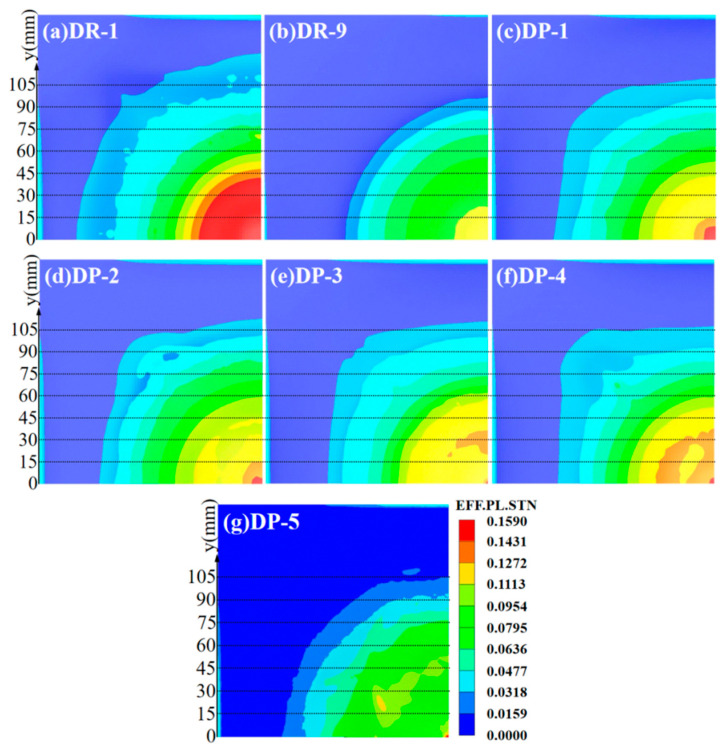
Effective plastic strain of back panels at t = 1000 μs: (**a**) DR-1; (**b**) DR-9; (**c**) DP-1; (**d**) DP-2; (**e**) DP-3; (**f**) DP-4; (**g**) DP-5.

The contour maps of effective plastic strain for the reference panels and the back panels of sandwich panels with different arrangement strategies are shown in Figure 14. In the reference panels, the DR-1 back panel had the largest range of effective plastic strain, reaching 0.0318 and 0.1431, with y-values of 126.05 mm and 41.91 mm. This indicated the weakest blast resistance. On the other hand, the effective plastic strain of the DR-9 back panel did not reach 0.1431, and the range with 0.0318 had a y-value of 96.41 mm. It indicated a stronger blast resistance than those of DP-1, DP-2, DP-3, and DP-4. Unlike the other panels, the DP-5 back panel still had a large area with effective plastic strain which was lower than 0.0159, and only a very small region with effective plastic strain, reaching 0.1272. This indicated the best blast resistance. To quantitatively assess the blast resistance of the back panels, the y-values where the effective plastic strain reached 0.1431 for DP-1 to DP-4 were compared. The DP-3 and DP-4 back panels had very small ranges of effective plastic strain, reaching 0.1431, with y-values of 2.09 mm and 4.23 mm. This indicated stronger blast resistance. The DP-2 and DP-1 back panels had slightly larger ranges, with y-values of 8.50 mm and 10.63 mm. This indicated a slightly weaker blast resistance. The ranking of blast resistance for the back panels is as follows: DP-5 > DR-9 > DP-3 > DP-4 > DP-2 > DP-1 > DR-1.

In short, smaller deflections and weaker localization will result in smaller effective plastic strains in the front panel. The limited effect of weaker localization on effective plastic strains in the back panel is primarily determined by the deflection of the panel.

#### 3.1.4. Analysis of Energy Absorption Characteristics

One crucial role of the core layer in a sandwich panel is to effectively absorb energy. Therefore, studying the energy absorption capability of the sandwich panel under air blast loading holds significant importance. To delve into this capability, researchers employed the energy absorption ($E_{a}$) and specific energy absorption (*SEA*) [25] indicators as the discussion means. The specific energy absorption (*SEA*) is defined as Equation (4):

(4) $$SEA=\frac{E_{a}}{\rho Ah}$$

In the energy absorption analysis, $E_{a}$ represents the absorbed energy, while ρ, *A*, and *h* denote the material density, area, and thickness of the structure, respectively.

**Figure 15:**
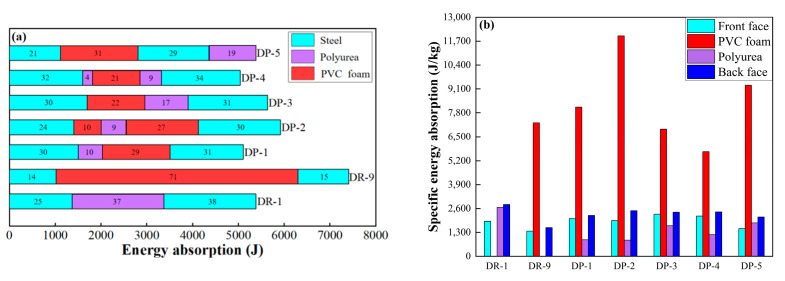
Energy absorption and specific energy absorption of the components of the sandwich panels with different arrangement strategies: (**a**) Energy absorption; (**b**) Specific energy absorption.

The energy absorption characteristics of the components of the sandwich panel were illustrated using a stacked bar graph, as shown in Figure 15a. The stacked bar graph distinguishes the materials of the panel and core layers with different colors, and the order from left to right shows front panel, core layer, and back panel. The proportion of energy absorption contribution for each component of the sandwich panel was quantified. DR-9, as the reference board, exhibited higher energy absorption by PVC foam than the polyurea in DR-1. However, the energy absorbed by the panel was lower in DR-9 than that in DR-1. Comparing DP-1, DP-2, DP-3, DP-4, and DP-5, the DP-2 sandwich panel absorbed the highest amount of energy, while DP-4 absorbed the least. The use of polyurea as the first core layer resulted in lower energy absorption by the sandwich panel, as seen in DP-1 and DP-4. In DP-2 and DP-1, it can be observed that PVC foam absorbed more energy than polyurea. Both polyurea and PVC foam, as core layers adjacent to the back panel, can absorb more energy. In DP-5, the proportion of energy absorbed by polyurea and PVC foam reached up to 50%, and the front panel absorbed the least energy but exhibited higher effective plastic strain. The higher localization of the front panel led to a concentration of strain in the central region and reduced the energy absorption potential of the front panel.

The relative energy absorption characteristics of the components of the sandwich panel are illustrated by using a bar graph, as shown in Figure 15b. The specific energy absorption capability of PVC foam per unit mass was higher than that of 304L stainless steel and polyurea. DP-2 exhibited the strongest energy absorption capability for PVC foam, and cutting PVC foam into two sections and adding a polyurea interplay can enhance its energy absorption capability. Its specific energy absorption is 64.7% higher than that of the reference panel DR-9. When PVC foam was adjacent to both the front and back panels (DP-5), or when it was adjacent to the back panel only (DP-1), it experienced more effective compression. On the other hand, the presence of a polyurea core layer between PVC foam and the back panel (DP-3), or the presence of a polyurea core layer between PVC foam and both the front and back panels (DP-4), reduced the compression of PVC foam. Polyurea adjacent to the back panel also exhibited stronger energy absorption capability (DR-1 and DP-3). However, polyurea is not an ideal energy-absorbing material, and the specific energy absorption of polyurea core layers in DP-1 to DP-4 was lower than that of the front and back panels.

#### 3.1.5. Pressure Analysis of Shock Waves

**Figure 16:**
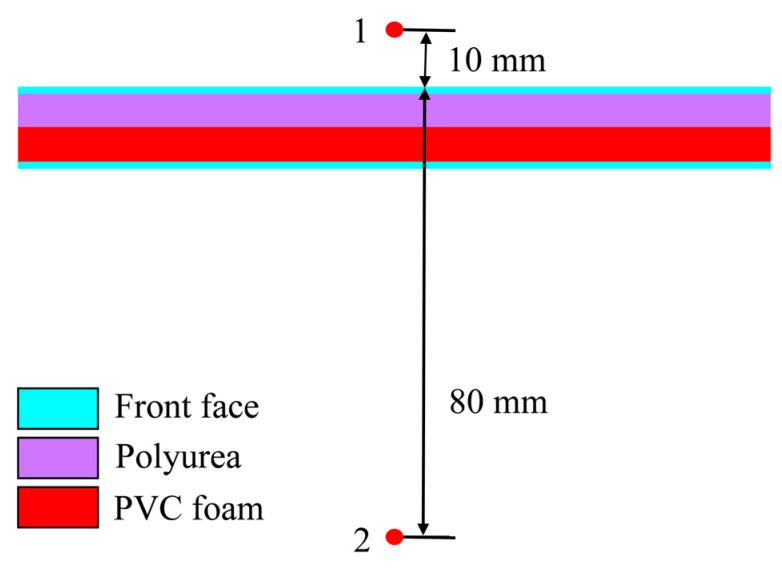
Placement of shock wave measurement points.

The blast resistance of sandwich panels is reflected in their structural strength, and in their capability to attenuate shock waves. Figure 16 shows the placement of two measurement points for reflecting overpressure (Point 1) at 10 mm from the front surface and attenuated overpressure (Point 2) at 80 mm behind the panel.

**Figure 17:**
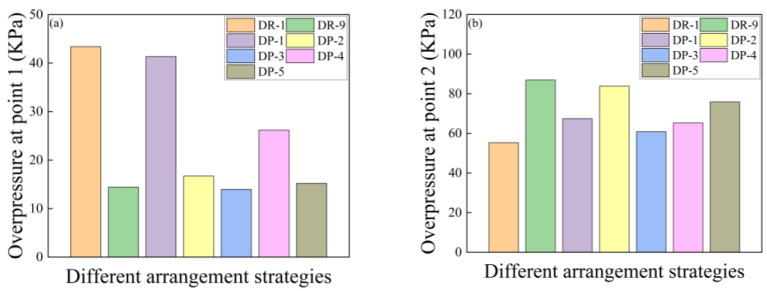
Overpressure peaks at points of sandwich panels with different arrangement strategies: (**a**) Reflected overpressure peaks at point 1; (**b**) Overpressure peaks at point 2.

Figure 17a,b respectively show the peak reflected overpressure at measurement point 1 and measurement point 2 for the reference board and the sandwich board with different arrangement strategies. Compared to the reference panel, it was observed that the reflected peak overpressure at Point 1 for DR-1 was 198.7% more than that of DR-9. DP-1 and DP-4 had reflected peak overpressure at Point 1 that was 186.4% and 81.9% more than that of DR-9, respectively. DR-1 with an 8 mm thick polyurea core had a reflected peak overpressure at Point 1 that was only 4.3% higher than that of DP-1 with a 6 mm thick polyurea core. On the other hand, the arrangement strategies of DP-2 and DP-5 showed lower increases in reflected peak overpressure at Point 1, which were only 15.2% and 5.2% more than that of DR-9, respectively. Having more core layers does not create better shock wave attenuation. DP-3 even showed a slight decrease in the reflected peak overpressure.

At Point 2, which was the measurement point for attenuated shock wave, DR-1 showed a peak overpressure that was 36.5% less than that of DR-9. All the sandwich panel configurations, from DP-1 to DP-5, exhibited a certain level of attenuation in the peak overpressure of the shock wave at Point 2. Among them, DP-3 showed the best attenuation effect on the shock wave, which was 29.8% less than that of DR-9. DP-2 demonstrated the poorest attenuation effect on the shock wave, only 3.4% less than that of DR-9.

In summary, polyurea core materials with the same surface density have stronger capability to reflect shock waves and better shock wave attenuation capability, compared to PVC foam. Panels with less shock wave reflection can absorb more energy. Improving the panel’s capability to reflect shock waves is one way to enhance its resistance to air blast loading. Additionally, directly spraying on the front panel can enhance the panel’s capability to reflect shock waves. Thicker polyurea layers provide better reflection effects when they do not exceed 6 mm in thickness; the increase in reflection effectiveness becomes smaller when the thickness exceeds 6 mm.

Considering the overall structural strength and shock wave attenuation capability of sandwich panels with different arrangement strategies, the DP-3 sandwich panel configuration provided effective protection for the back panel and effectively attenuates the shock wave, which makes it the optimal choice for blast resistance. Although the DR-1 panel, which only consists of polyurea as the core layer, exhibited the strongest ability to attenuate shock waves, it provided poor protection for the back panel. On the other hand, while DP-5 and DR-9 panel configurations that utilized only PVC foam as the core layer, offered better protection for the back panel, their ability to attenuate shock waves was comparatively weaker.

#### 3.1.6. Blast r3.1.6 Blast Resistance Performance Assessment

To comprehensively compare the blast resistance capacities of sandwich panels with different arrangement strategies, factors such as the protection of the back panel, energy absorption capability, and shock wave attenuation capability were taken into consideration. The following Equation (5) was used to calculate the blast resistance of the sandwich panel:

(5) $$G=100\times \left[\frac{\left(\bar{w_{0}}-w_{0}\right)}{\bar{w_{0}}}+\frac{\left(E-\bar{E}\right)}{\bar{E}}+\frac{\left(\bar{P}-P\right)}{\bar{P}}\right]$$

In the equation, *G* represents the score for the blast resistance capability of the sandwich panel. $w_{0}$ and $\bar{w_{0}}$ refer to the deflection values at the midpoint of the back panel for the sandwich panel and the average deflection values for different arrangement strategies of the sandwich panel, respectively. *E* and $\bar{E\;}$ signify the energy absorbed by the sandwich panel and the average energy absorption values for different arrangement strategies of the sandwich panel, respectively. *P* and $\bar{P}$ denote the peak overpressure values at point 2 of the sandwich panel and the average peak overpressure values at point 2 for different arrangement strategies of the sandwich panel, respectively. The calculated results for the blast resistance scores of different arrangement strategies of the sandwich panel are shown in Table 6.

The arrangement strategy of DP-3 (S-F-P-S) sandwich panel has the highest blast resistance score, 16.72. This strategy provided good protection for the back panel, with strong energy absorption capability and the ability to attenuate shockwaves to a large extent. It therefore had the best blast resistance.

### 3.2. Analysis of Numerical Simulation Results of Different Quality Ratios

#### 3.2.1. Analysis of Deformation Characteristics of the Panel

To investigate the θ values fitted with different mass ratios, refer to Table 7.

As the mass ratio of polyurea decreased and the ratio of PVC foam increased, the θ value of the front panel initially increased and reached a peak at DR-5, and then stabilized, but slightly decreased at DR-9. The thickness of the PVC foam played a dominant role in providing greater freedom of movement for the deformation of the front panel before reaching a thickness of 16 mm. At this point, thinner polyurea and thicker PVC foam both resulted in a higher degree of localization in the front panel. However, when the thickness of the PVC foam exceeded 16 mm, the increased bending resistance of the thicker PVC foam actually hindered further deformation in the central area of the front panel. Therefore, from DR-6 to DR-9, thinning the polyurea and thickening the PVC foam showed minimal changes in the degree of localization of the front panel, or even a slight weakening at DR-9.

The degree of localization in the back panel was less influenced by the mass ratio of polyurea to PVC foam, and the θ values are mostly concentrated around 7. It can be seen that the back panel still experienced overall tensile deformation as the main mode. The overall tensile deformation of the thicker polyurea, along with the buffering effect and higher bending resistance of the thicker PVC foam, prevented localized deformation in the back panel. In the DR-5 sandwich panel, where both the polyurea and PVC foam thicknesses were moderate, it resulted in a higher degree of localization in the back panel, with a θ value of 9.09.

#### 3.2.2. Panel Midpoint Deflection Analysis

**Figure 18:**
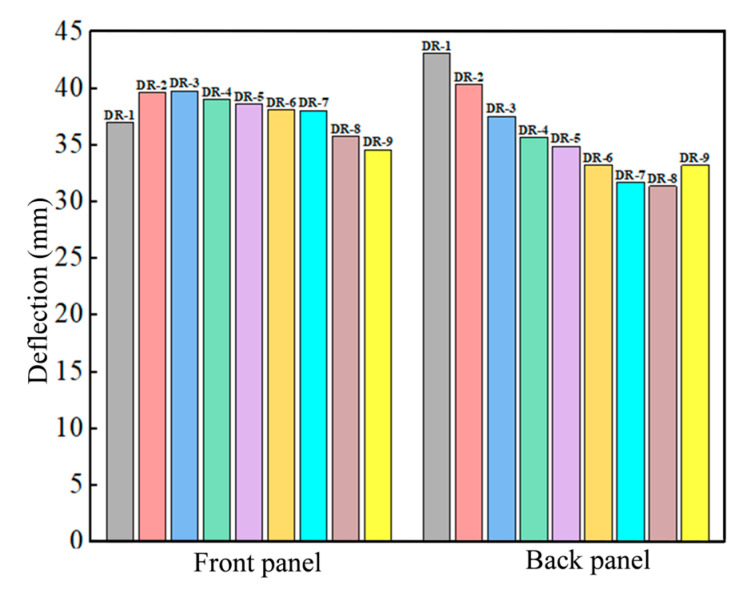
Midpoint deflections of sandwich panels with different mass ratios.

The average of the peak deflections at the midpoint of the panels was taken as the final deflection, as shown in Figure 18, for various mass ratios in sandwich panels. As the mass ratio of PVC foam increases, the deflection at the midpoint of the front panel initially increases and then decreases. The DR-3 front panel had the highest deflection at its midpoint. When the thickness of PVC foam was less than 8 mm, its buffering effect effectively reduced the support force on the front panel from the core layer and led to an increase in deflection at the midpoint of the front panel. However, the deflection at the midpoint of the DR-3 front panel was 3.2% more than DR-2. It indicated that the buffering effect of 8 mm PVC foam was only slightly improved compared to 4 mm PVC foam, and thicker PVC foam provided stronger bending resistance that limited the displacement of the front panel. When the thickness of PVC foam exceeded 8 mm, the front panel exhibited a stronger localization. At this point, the stronger bending resistance of thicker PVC foam became the dominant factor and led to a decrease in deflection at the midpoint of the front panel as the thickness of PVC foam increased.

As the mass ratio of PVC foam increased, the deflection at the midpoint of the back panel continuously decreased, but there was a slight increase in DR-9. The deflection at the midpoint of the DR-2 and DR-3 back panels was 6.3% and 12.8% less than that of DR-1, respectively. This indicated a significant decrease in deflection. Thicker PVC foam provided better cushioning, offering stronger resistance to bending, and led to a significant reduction in deflection at the midpoint of the back panel. The decrease in deflection at the midpoint of the back panels for panels from DR-4 to DR-8 was relatively smaller, as the improvement in cushioning effect was limited after the thickness of PVC foam exceeded 8 mm. However, the reduction of the polyurea thickness increased the energy which was transferred from the shock wave to the sandwich panel. This is the reason why the deflection at the midpoint of the DR-9 back panel was 6% more than that of DR-8.

#### 3.2.3. Effective Plastic Strain Analysis of Panel

**Figure 19:**
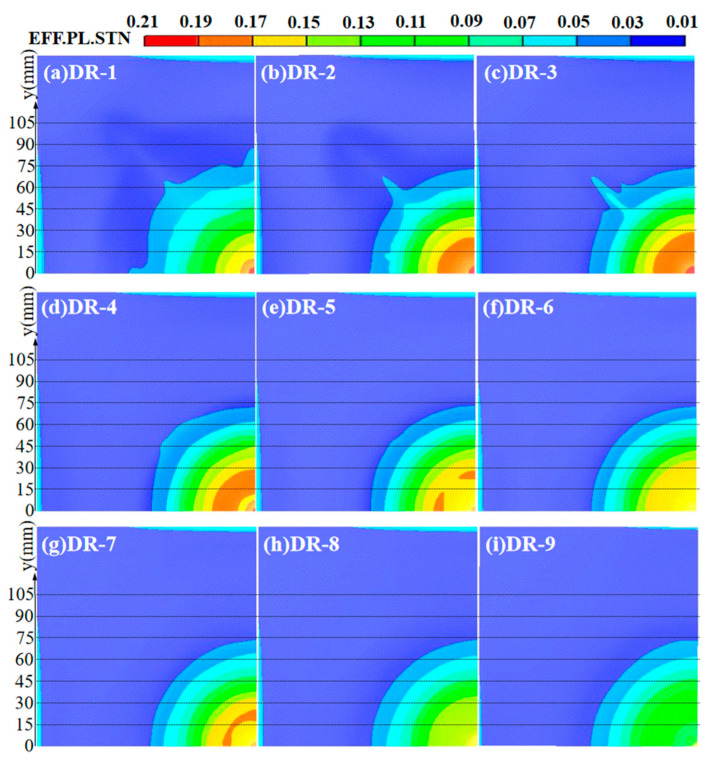
Effective plastic strain of front panels: (**a**) DR-1; (**b**) DR-2; (**c**) DR-3; (**d**) DR-4; (**e**) DR-5; (**f**) DR-6; (**g**) DR-7; (**h**) DR-8; (**i**) DR-9.

The contour map of equivalent plastic strain on the front panel of different mass ratio sandwich panels is shown in Figure 19. When the equivalent plastic stain was over 0.17, the range was relatively larger for the front panels from DR-2 to DR-4. This indicated a weaker blast resistance. Comparing the range of equivalent plastic strain exceeding 0.19, the DR-3 front panel had the weakest blast resistance, while the DR-4 front panel had the strongest. The front panels of DR-1, DR-5, and DR-7 had a smaller range when the equivalent plastic strain exceeded 0.17, but a larger range when it exceeded 0.15. Their blast resistance was moderate. The y-value of the DR-1 front panel when it reached an equivalent plastic strain of 0.15 was only 20.9 mm, much smaller than that of DR-5 and DR-7. The range when the equivalent plastic strain reached 0.17 in the central region is also small, indicating stronger blast resistance than DR-5 and DR-7. On the other hand, for the DR-7 front panel, both the y-values corresponding to equivalent plastic strains of 0.17 and 0.15 are smaller than that of DR-5, indicating slightly stronger blast resistance than DR-5. The maximum equivalent plastic strain of the DR-6, DR-8, and DR-9 front panels was less than 0.17, which indicated the best blast resistance. A y-value of 42.7 mm corresponded to the DR-6 front panel when an equivalent plastic strain exceeded 0.15, indicating a slightly weaker blast resistance. The y-values of the DR-8 front panel corresponding to equivalent plastic strains which exceeded 0.15 and 0.13 were 2.2 mm and 34.7 mm, respectively, indicating a stronger blast resistance than DR-6. The y-value of 2.1 mm corresponded to the DR-9 front panel when an equivalent plastic strain exceeded 0.13. Among the different mass ratio sandwich panels, the DR-9 front panel had the smallest equivalent plastic strain and the best blast resistance. The relationship between the equivalent plastic strain and panel deflection on the front panel of different mass ratio sandwich panels is closely related. Larger deflections are usually accompanied by larger equivalent plastic strains, indicating weaker blast resistance. However, the effective plastic strain of the DR-1 and DR-6 front panels was not solely determined by deflection. The ranking of front panel blast resistance is as follows: DR-9 > DR-8 > DR-6 > DR-1 > DR-7 > DR-5 > DR-4 > DR-2 > DR-3.

**Figure 20:**
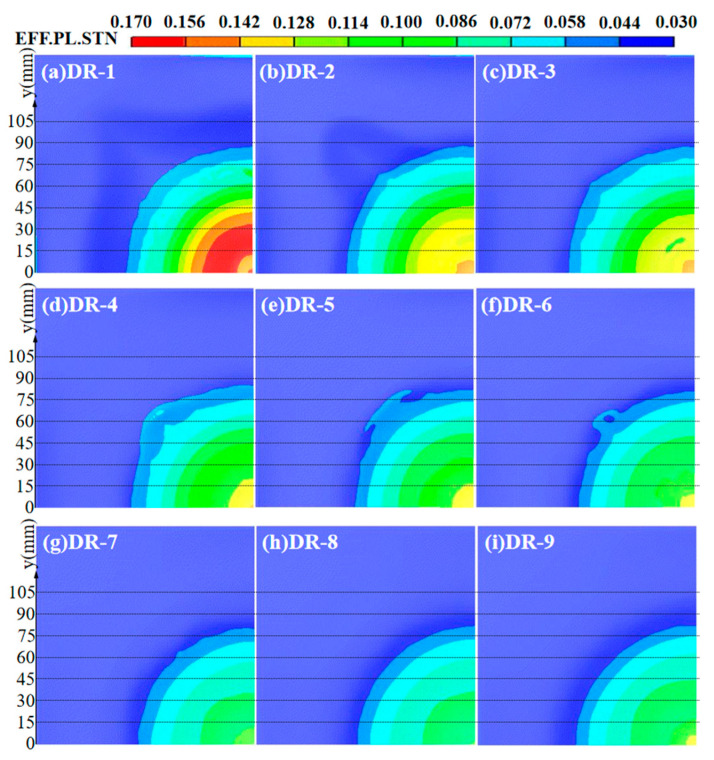
Effective plastic strain of back panels: (**a**) DR-1; (**b**) DR-2; (**c**) DR-3; (**d**) DR-4; (**e**) DR-5; (**f**) DR-6; (**g**) DR-7; (**h**) DR-8; (**i**) DR-9.

The strain contour map of the back panel of different mass ratio sandwich panels is shown in Figure 20. By comparing the maximum effective plastic strain and its area in the central region of the back panels, it was found that the blast resistance of the back panel increased with the increase of PVC foam mass ratio. However, the blast resistance of the DR-9 back panel, which consisted of only PVC foam core, was slightly weaker than that of DR-7 and DR-8. The effective plastic strain and blast resistance of the back panel had the same trend as deflection. Greater deflection of the back panel indicates larger effective plastic strain and weaker blast resistance. The ranking of blast resistance for the back panel is as follows: DR-8 > DR-7 > DR-9 > DR-6 > DR-5 > DR-4 > DR-3 > DR-2 > DR-1.

In summary, thicker PVC foam provides good protection for the front and back panels due to its cushioning effect and greater bending resistance. The polyurea coating applied to the front panel also enhances the blast resistance of the front and back panels by reducing the energy transferred to the sandwich panels by the shock wave and providing support to the front panel.

#### 3.2.4. Analysis of Energy Absorption Characteristics

**Figure 21:**
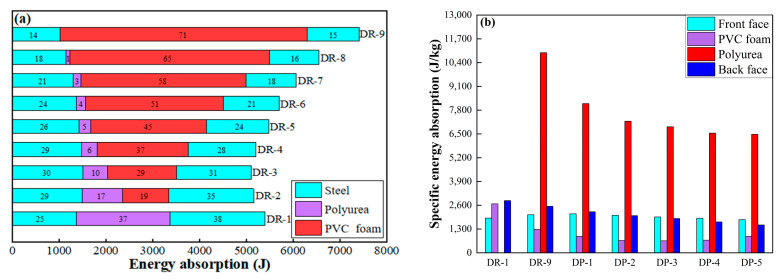
Energy absorption and specific energy absorption of the components of the sandwich panels with different mass ratios: (**a**) Energy absorption; (**b**) Specific energy absorption.

Figure 21a represents the energy absorption characteristics of each component of the sandwich panel. As the mass ratio of PVC foam increased, the total energy absorbed by the sandwich panel initially decreased and then increased. DR-3 had a minimum total energy absorption of 5106.9 J, while DR-9 had a maximum total energy absorption of 7413.4 J. The energy absorbed by both PVC foam and polyurea is positively correlated with their mass ratios. Higher quality ratios of PVC foam and polyurea enable them to absorb more energy.

The specific energy absorption characteristics of each component of the sandwich panel are depicted in Figure 21b. Both the front and back panels showed a consistent trend with their deflection. Polyurea exhibited a relatively weak energy absorption capability per unit mass, and only DR-1 showed a higher specific energy absorption than the front panel. The specific energy absorption of polyurea is influenced by the presence of PVC foam. For instance, the introduction of 4 mm thick PVC foam in DR-2 resulted in a significant 50.8% decrease in specific energy absorption compared to DR-1. With the increase of PVC foam quality ratio, the specific energy absorption of PVC foam first decreased and then increased. The specific energy absorption of PVC foam in the DR-7 sandwich panel was the lowest, only 59.5% of DR-2. The specific energy absorption of PVC foam is influenced by both its own thickness and the thickness of polyurea. Thinner PVC foam or thinner polyurea will result in higher specific energy absorption of PVC foam.

#### 3.2.5. Pressure Analysis of Shock Wave

**Figure 22:**
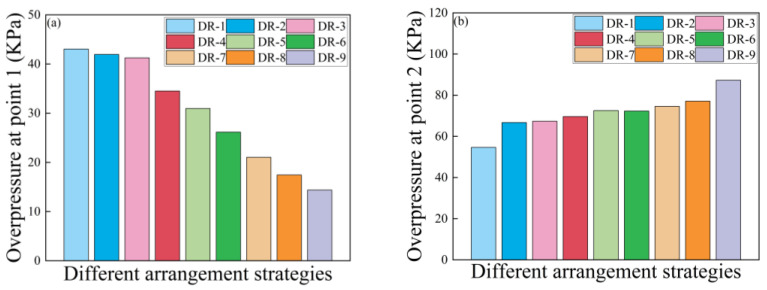
Overpressure peaks at measurement points of sandwich panels with different arrangement strategies: (**a**) Reflected overpressure peaks at point 1; (**b**) Overpressure peaks at point 2.

Figure 22a presents the peak reflection overpressure at measurement Point 1 for sandwich panels with different mass ratios. The reflection overpressure at measurement Point 1 decreased as the quality ratio of the polyurea decreased. Although sandwich panels with thicker polyurea in panels from DR-1 to DR-3 showed better reflection effects on the shock wave, the thicker polyurea coating approached rigid reflection, and the enhancement effect was not significant.

Figure 22b displays the peak overpressure at measurement point 2 for sandwich panels with different mass ratios. As the polyurea mass ratio decreased, the peak overpressure of the shock wave at measurement Point 2 continued to increase. This indicates that the attenuation capability of the shock wave decreased as the polyurea mass ratio decreased. Sandwich panels with a POLYUREA/PVC foam composite core layer (DR-2 to DR-8) exhibited significantly improved attenuation capability in comparison with DR-9. Sandwich panels from DR-2 to DR-8 exhibited structural strength and shock wave attenuation capability that fell between the values observed in DR-1 and DR-9.

By combining the structural strength of sandwich panels with varying mass ratios under air blast loading and their shock wave attenuation capability, the calculation results of the blast-resistance ability scores of sandwich panels with different mass ratios are shown in Table 8. The ratio strategy of DR-8 ($m_{p}$: $m_{f}$ = 1:7) sandwich panel have the highest blast resistance score of 17.68. It significantly enhanced the protection capability of the back panel, and at the same surface density, DR-8 showed a balanced and excellent blast resistance capability.

## 4. Conclusions

Numerical simulation was employed to investigate the dynamic response of PVC foam/polyurea composite sandwich panels subjected to close blast loading, with particular focus on the impact of different arrangement strategies and mass ratios on the blast resistance of the panels under equal surface density. The obtained findings can be summarized as follows:

(1) The process of deformation is divided into three stages: 1. Fluid-structure coupling interaction; 2. Sandwich compression; 3. Structural dynamic response stage.
(2) The front panel has strong localization characteristics while the back panel is dominated by large overall deformation. The front panel with small localization degree has a large deflection. The deflection of the back panel can evaluate the structural strength of sandwich panel under blast loading.
(3) Polyurea possesses good shock wave attenuation capability, and the thicker the polyurea, the stronger the laminate’s ability to reflect shock waves.
(4) The anti-explosion capabilities of laminates with different arrangement strategies and mass ratios were comprehensively compared, revealing that the DP-3 (S-F-P-S) arrangement strategy and the DR-8 ($m_{p}$: $m_{f}$ = 1:7) mass ratio exhibit the best anti-explosion performance.

This article addresses the impact of the arrangement of PVC foam and polyurea on anti-explosion performance, providing reference for future sandwich board design. This study used a balanced evaluation method to assess different sandwich structures, and the evaluation formula can be weighted for different application scenarios to select the most suitable arrangement method. However, relying solely on simulation, this approach can only provide qualitative analysis, and the parameters used in the simulation may not be practical when there are changes in loading conditions. Therefore, it is necessary to employ experimental methods for verification. Furthermore, this study only focused on an 80 g TNT equivalent, which is equivalent to a hand grenade, without considering the coupling effect between fragments and shock waves. Hence, further research is needed to enhance the practicality of the anti-explosion laminate.

## Figures and Tables

**Table 1:** Material properties of PVC foam (H250).

Mass Density/kg/m^3^	Compressive Strength/MPa	Compression Modulus/MPa	Tensile Strength/MPa	Tensile Modulus/MPa	Shear Strength/MPa	Shear Modulus/MPa
250	7.2	400	9.2	320	4.5	97

**Table 2:** Experimental conditions.

Name	Front Panel Thickness (mm)	Back Panel Thickness (mm)	Core Layer	Core Layer Thickness (mm)	Panel Size (mm)	Surface Density (kg/m^3^)
USP-1	1.38	1.38	H250	14	300 × 288	25.17
USP-2	0.9	1.38	H250	14	300 × 288	21.4
USP-3	1.8	1.38	H250	14	300 × 288	28.46
USP-4	1.38	0.9	H250	14	300 × 288	21.4
USP-5	1.38	1.8	H250	14	300 × 288	28.46
USP-6	1.38	1.38	H250	9	300 × 288	23.92
USP-7	1.38	1.38	H250	20	300 × 288	26.67

**Table 3:** Material properties and Johnson–Cook parameters of 304 stainless steel.

Mass Density ρ/(kg/m^3^)	Volume Modulus K/GPa	Shear Modulus G/GPa	Yield Strength A/MPa	Hardening Constant B/MPa	Hardening Index *n*
7900	166.7	77.0	310	1000	0.65
**Strain Rate Constant *c***	**Thermal Softening Index *m***	**Room Temperature *Tr*/K**	**Melt Temperature *Tm*/K**	**Effective Plastic Strain Rate *ε*/s^−1^**	
0.07	1	293	1673	1	

**Table 4:** Detailed information of the sandwich panels.

Name	Material Arrangement	t1	t2	t3	t4	t5	Surface Density	Blast Distance
	(Front → Back)	(mm)	(mm)	(mm)	(mm)	(mm)	(kg/m^2^)	(mm)
DP-1	S-P-F-S	1	6	8	1		24.92	50
DP-2	S-F-P-F-S	1	4	6	4	1	24.92	50
DP-3	S-F-P-S	1	8	6	1		24.92	50
DP-4	S-P-F-P-S	1	3	8	3	1	24.92	50
DP-5	S-F-S-P	1	8	1	6		24.92	50
DR-1	S-P-S	1	8	1			24.96	50
DR-2	S-P-F-S	1	7	4	1		24.94	50
DR-3	S-P-F-S	1	6	8	1		24.92	50
DR-4	S-P-F-S	1	5	12	1		24.90	50
DR-5	S-P-F-S	1	4	16	1		24.88	50
DR-6	S-P-F-S	1	3	20	1		24.86	50
DR-7	S-P-F-S	1	2	24	1		24.84	50
DR-8	S-P-F-S	1	1	28	1		24.82	50
DR-9	S-F-S	1	32	1			24.80	50

**Table 5:** Fitted results of panel deformation with different arrangement strategies.

	DR-1	DR-9	DP-1	DP-2	DP-3	DP-4	DP-5
Front panel	8.90	13.10	10.75	18.54	14.46	12.07	21.85
Back panel	7.29	6.93	7.04	8.36	6.76	7.04	10.61

**Table 6:** Blast resistance scores for sandwich panels with different arrangement strategies.

	DP-1	DP-2	DP-3	DP-4	DP-5
Deflection score at the midpoint of the back panel	−4.25	−5.54	−1.08	−0.39	11.25
Energy absorption score	−5.72	9.28	4.07	−6.95	−0.68
Shockwave attenuation score	4.80	−18.72	13.73	7.58	−7.39
Total	−5.17	−14.98	16.72	0.24	3.18

**Table 7:** Fitted results of panel deformation with different mass ratios.

	DR-1	DR-2	DR-3	DR-4	DR-5	DR-6	DR-7	DR-8	DR-9
The front panel	8.90	9.61	10.75	12.17	15.56	15.33	15.50	15.53	13.10
The back panel	7.29	7.01	7.04	7.67	9.09	7.65	7.24	7.72	6.93

**Table 8:** Blast resistance scores for sandwich panels with different ratio strategies.

	DR-1	DR-2	DR-3	DR-4	DR-5	DR-6	DR-7	DR-8	DR-9
Deflection score at the midpoint of the back panel	−20.75	−13.10	−5.24	0.00	2.20	6.83	11.12	12.09	6.86
Energy absorption score	−6.86	−10.88	−11.71	−10.04	−5.35	−1.44	4.87	13.25	28.16
Shock wave attenuation score	22.84	6.35	5.73	2.65	−1.77	−1.84	−4.52	−7.66	−21.78
Total	−4.77	−17.63	−11.22	−7.39	−4.92	3.55	11.47	17.68	13.24

## Data Availability

Data are contained within the article.
